# ﻿Genotyping the phenotypic diversity in Aegean *Natrixnatrixmoreotica* (Bedriaga, 1882) (Reptilia, Serpentes, Natricidae)

**DOI:** 10.3897/zookeys.1169.104594

**Published:** 2023-07-13

**Authors:** Daniel Jablonski, Elias Tzoras, Alexios Panagiotopoulos, Marika Asztalos, Uwe Fritz

**Affiliations:** 1 Department of Zoology, Comenius University in Bratislava, Ilkovičova 6, Mlynská dolina, 842 15, Bratislava, Slovakia Comenius University in Bratislava Bratislava Slovakia; 2 Patras, 264 42 Achaia, Greece Unaffiliated Patras Greece; 3 Section of Animal Biology, Department of Biology, University of Patras, 26500 Patras, Greece University of Patras Patras Greece; 4 Museum of Zoology (Museum für Tierkunde), Senckenberg Dresden, A. B. Meyer Building, 01109 Dresden, Germany Museum of Zoology Dresden Germany

**Keywords:** Cyprus, Greece, melanism, *picturata* morphotype, *picturata* morphotype, *schweizeri* morphotype, taxonomy

## Abstract

We examined the mitochondrial identity of Aegean *Natrixnatrixmoreotica* representing different morphotypes, with a focus on new material from Milos and Skyros. We found no correlation between distinct morphotypes and mitochondrial identity. Our results support that grass snake populations are polyphenetic and that southern subspecies, including island populations, show a higher variability than northern ones.

Mediterranean islands are well known for their rich biodiversity with many endemic taxa. This diversity is particularly evident in the Aegean region, which has undergone a complex geological history shaping its biota (Poulakakis et al. 2015). Many Aegean islands have been isolated since the Miocene and, as a result, harbour endemic taxa. This includes, for example, the amphibians *Lyciasalamandrahelverseni* (Pieper, 1963) and *Bufotesviridisdionysi* Dufresnes, Probonas & Strachinis, 2020 and the reptiles *Podarciscretensis* (Wettstein, 1952), *Mediodactylusbartoni* (Štěpánek, 1934), and *Macroviperaschweizeri* (Werner, 1935). However, the diversity of the Mediterranean island herpetofauna has traditionally often been overestimated. During the 19^th^ and the first half of the 20^th^ century, many subspecies were described that reflect merely local phenotypes (Mertens and Wermuth 1960), which are not even necessarily restricted to the islands from where the taxa were described. Prime examples for this taxonomic inflation are the many invalid subspecies of island lizards (e.g., *P.siculus*, see Henle and Klaver 1986; Podnar et al. 2005). Also, some Aegean island populations of the grass snake *Natrixnatrix* (Linnaeus, 1758) have been described as endemic subspecies, e.g., *Tropidonotusnatrixsyrae* Hecht, 1930 from Syros or *Tropidonotusnatrixdystiensis* Hecht, 1930 from Euboea (Fig. 1). Only three decades ago, another subspecies, *Natrixnatrixfusca* Cattaneo, 1990, was erected from Kea (Cattaneo 1990). None of these subspecies is currently recognized and these taxa are understood to represent phenotypic variation (Fritz and Schmidtler 2020; Asztalos et al. 2021a).

**Figure 1.**
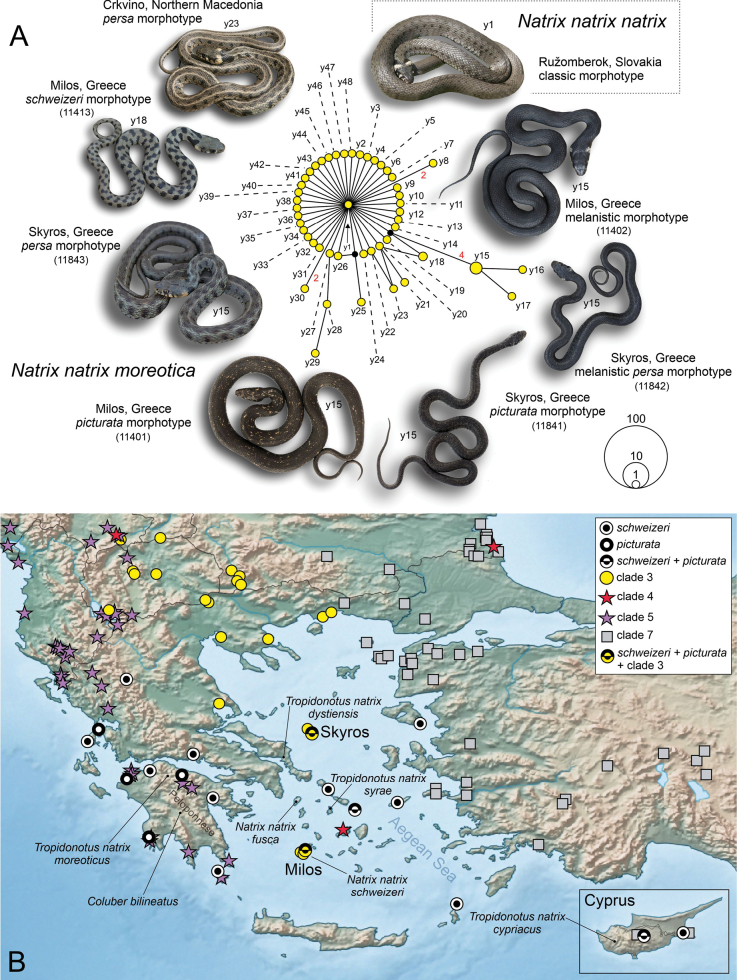
**A** parsimony network for 54 ND4+tRNAs sequences of clade 3 (“yellow lineage” of Kindler et al. 2013, 2017) of *Natrixnatrix.* Photos show different grass snake phenotypes. Symbol size reflects haplotype frequency. Small black circles are missing node haplotypes; each line connecting two haplotypes corresponds to one mutation step, if not otherwise indicated by red numbers along lines. Haplotype terminology for *N.natrix* follows Kindler et al. (2013) (photos: D. Jablonski, E. Tzoras) **B** distribution of mitochondrial clades (from Kindler et al. 2013; Asztalos et al. 2021a) and of the *picturata* and *schweizeri* morphotypes in the the Aegean region and Cyprus (see details in Suppl. material 1: table S1). In addition, the type localities (see Fritz and Schmidtler 2020) of local taxa are indicated.

Milos, a small Aegean island with limited freshwater resources, is home to grass snakes that are less aquatic than elsewhere (Kratzer 1974). This population exhibits considerable phenotypic variation, and one of the local phenotypes led to the description of the subspecies *Natrixnatrixschweizeri* Müller, 1932, thought to be endemic to Milos and the neighbouring islands Kimolos and Polyaegos (Kabisch 1999). This taxon was originally erected for grass snakes bearing large dark blotches on the dorsal and lateral body (Müller 1932). Recent genetic studies by Kindler et al. (2013, 2017) and Asztalos et al. (2021a) have revealed that grass snakes from Milos possess mitochondrial haplotypes of the so-called “yellow lineage” (clade 3) of *N.natrix*, which is found throughout central-western Europe and parts of the Balkans. Following the subspecies concept and the criteria outlined in Kindler and Fritz (2018), Asztalos et al. (2021a) concluded that all grass snakes from the southern Balkans, western Turkey, and Cyprus represent the subspecies *Natrixnatrixmoreotica* (Bedriaga, 1882), which implicitly included all Aegean populations, i.e., also grass snakes from Milos. However, Asztalos et al. (2021a) did not provide information about the phenotypes of the genetically studied snakes.

Besides the “*schweizeri* morphotype,” melanistic grass snakes and the “*picturata* morphotype” are known to occur on Milos (Kreiner 2007). Representatives of the *picturata* morphotype are best described as melanistic grass snakes with many small light speckles (Kabisch 1999). All of these morphotypes have also been recorded outside of Milos, and they are known to coexist on other islands, such as Mykonos and Cyprus, in part together with additional phenotypes, like the striped “*picturata* morphotype” (Fig. 1; Wiedl and Böhme 1992; Covaciu-Marcov et al. 2006; Baier and Wiedl 2010; Cattaneo 2010; Bogaerts et al. 2018; Zotos et al. 2021; Fănaru et al. 2022; Fritz and Ihlow 2022).

In the subspecies *N.n.moreotica*, several mitochondrial lineages with many individual haplotypes occur in close geographic proximity or even together (Fig. 1; Kindler et al. 2013, 2017; Asztalos et al. 2021a). Until now, there was no attempt made to examine whether the different phenotypes are possibly correlated with distinct mitochondrial lineages. In the present study, we examine whether grass snakes of the different phenotypes bear one and the same or different mitochondrial lineages or haplotypes.

During fieldwork in the western Aegean, specifically on Milos (Cyclades) and Skyros (Sporades), we collected buccal swabs from three grass snakes each from Milos and Skyros representing different phenotypes (Table 1). Colouration and pattern of the sampled snakes is shown in Figs 1 and 2. The snakes were observed in various types of freshwater habitats (Suppl. material 1: fig. S1).

**Figure 2.**
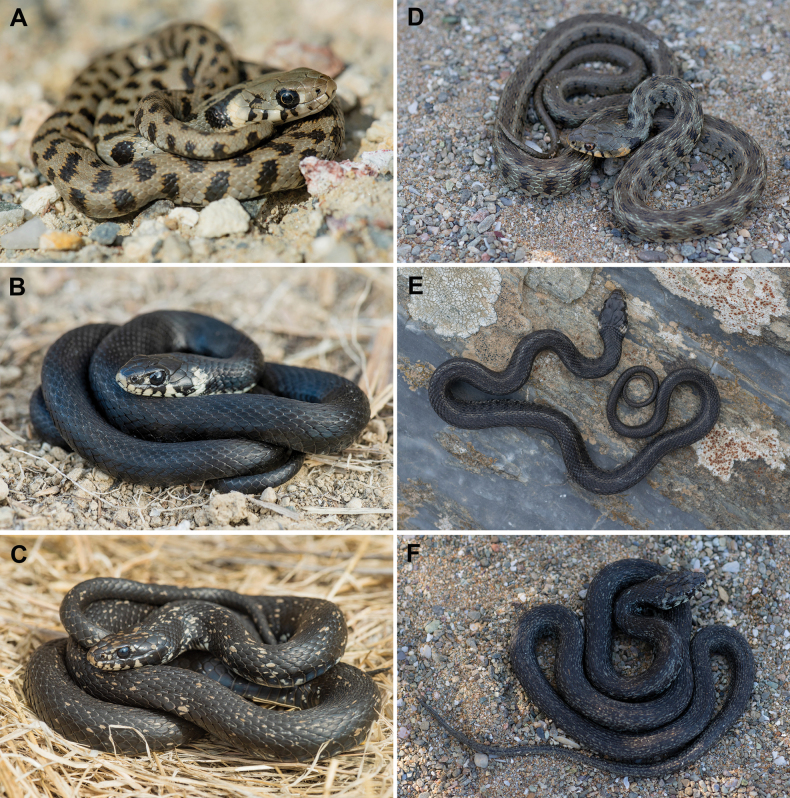
Morphotypes of *Natrixnatrix* from Milos and Skyros on natural ground **A***schweizeri* morphotype from Milos (11413) **B** melanistic morphotype from Milos (11402) **C***picturata* morphotype from Milos (11401) **D***picturata* morphotype from Skyros (11843) **E** melanistic *picturata* morphotype from Skyros (11842) **F***picturata* morphotype from Skyros (11841). Photos: D. Jablonski (**A–C**), E. Tzoras (**D–F**).

**Table 1.** Studied grass snake samples.

Field number	Sampled specimen	Collection site	Phenotype	Haplotype
11401	Adult female	Milos, 36.7078°N, 24.4897°E, 7 m a.s.l.	“*picturata*”	y15
11402	Subadult male	Milos, 36.7078°N, 24.4897°E, 7 m a.s.l.	melanistic	y15
11413	Juvenile; unknown sex	Milos, 36.7233°N, 24.5297°E, 51 m a.s.l.	“*schweizeri*”	y18
11841	Adult; unknown sex	Skyros, 38.9569°N, 24.5049°E, 10 m a.s.l.	“*picturata*”	y15
11842	Juvenile; unknown sex	Skyros, 38.9569°N, 24.5049°E, 10 m a.s.l.	melanistic “*picturata*”	y15
11843	Adult; unknown sex	Skyros, 38.9569°N, 24.5049°E, 10 m a.s.l.	regular “*picturata*”	y15

The buccal swabs were stored in 96% ethanol until analysis. Genomic DNA was extracted using standard DNA isolation kits and the partial ND4 gene plus adjacent DNA coding for tRNAs (tRNA-His, tRNA-Ser, tRNA-Leu) was amplified and sequenced following Kindler et al. (2013). The resulting mtDNA sequences (866 bp) were compared to the previously published data set of Kindler et al. (2013, 2017), Schultze et al. (2019), and Asztalos et al. (2021a, 2021b) using BioEdit (Hall 1999). For previously identified haplotypes of clade 3 (“yellow lineage”) and our new sequences, a parsimony network was drawn using TCS 1.21 (Clement et al. 2000) with gaps coded as fifth character state and a connection limit of 50 steps.

To obtain additional insights in the genetic and morphological variation of grass snakes in the Aegean region, we combined previously published genetic data (Kindler et al. 2013; Asztalos et al. 2021a) with literature and citizen-science data (mainly iNaturalist) for the distinct morphotypes (Suppl. material 1: table S1). Our definition of morphotypes follows Fritz and Schmidtler (2020) and Fritz and Ihlow (2022).

All previously published (Kindler et al. 2013) and our new mtDNA sequences of *N.natrix* from Milos and Skyros represent clade 3. The four previously published sequences and five of our six new sequences correspond to haplotype y15 (ENA accession number LT839118). Our subadult female from Milos with the *schweizeri* morphotype represents haplotype y18 (LT839121). The two haplotypes are similar and differ by five mutation steps (Fig. 1). Haplotype y15 is also known from Bulgaria, North Macedonia, Greece, and Serbia, whereas y18 was previously only recorded in Bulgaria. Closely related haplotypes are also found in the region, in particular y16 from Greece and y17 from Bulgaria and North Macedonia. These findings do not support that the different morphotypes correlate with different mitochondrial lineages or haplotypes.

The *picturata* and *schweizeri* morphotypes are not restricted to the Aegean region but also occur in other parts of the distribution range of *N.natrix* (Fritz and Ihlow 2022). The *schweizeri* morphotype is known for a long time from Milos, but also from Cyprus (Kabisch 1999; Baier et al. 2009). On the latter island occurs another mitochondrial lineage (clade 7; Fig. 1B). Using iNaturalist data, Fritz and Ihlow (2022) recorded the *schweizeri* morphotype also in continental Greece, on the Peloponnese, and on Ikaria (Dodecanese Islands), about 170 km northeast (airline) of Milos (Fig. 1B), i.e., in regions where mitochondrial clades 5 and 7 occur. The occurrence of the *schweizeri* morphotype in snakes harbouring different mitochondrial lineages implies that phenotypic and mitochondrial identities are decoupled. The same is true for the rare *picturata* morphotype, which sporadically occurs across the distribution ranges of *N.natrix* and *N.helvetica* (Fig. 1; Fritz and Schmidtler 2020; Bruni et al. 2022; Fritz and Ihlow 2022). Baier et al. (2009) reported for one site on Cyprus (Xyliatos Dam) the syntopic occurrence of regularly coloured grass snakes, melanistic individuals, and representatives of the *picturata* morphotype in almost equal frequencies, supporting that grass snake populations are polyphenetic. It seems that southern subspecies, including island populations, show a higher variability than northern ones (Fritz and Ihlow 2022).

It is likely that environmental and ecological conditions contribute to these local differences (see also Bury et al. 2020; Bruni et al. 2022), but a genetic component and selection are also likely to act in concert. For instance, the complete lack of the striped *picturata* morphotype both in *N.helvetica* and all northern populations of *N.natrix* (Fritz and Ihlow 2022; Fritz et al. 2023) would be hard to explain without a genetic component. To obtain a better understanding of the morphological variability of grass snakes, a comprehensive comparative study would be needed, examining how environmental variables, genetic identity, sex, and habitat are correlated with the different morphotypes.

## ﻿Additional information

### Author ORCIDs

Daniel Jablonski https://orcid.org/0000-0002-5394-0114

Marika Asztalos https://orcid.org/0000-0002-4090-1159

Uwe Fritz https://orcid.org/0000-0002-6740-7214

### Data availability

All of the data that support the findings of this study are available in the main text or Supplementary Information.

## Supplementary materials

Supplementary material 1 Supplementary information Data type PDf file Explanation note figure S1. Habitats of grass snakes on Milos; table S1. Literature and citizen science records of the *picturata* and *schweizeri* morphotypes of *Natrixnatrix* in the Aegean region and Cyprus. https://binary.pensoft.net/file/877335This dataset is made available under the Open Database License (http://opendatacommons.org/licenses/odbl/1.0/). The Open Database License (ODbL) is a license agreement intended to allow users to freely share, modify, and use this Dataset while maintaining this same freedom for others, provided that the original source and author(s) are credited.Daniel Jablonski, Elias Tzoras, Alexios Panagiotopoulos, Marika Asztalos, Uwe Fritz
