# Supplementary material for: ﻿Genotyping the phenotypic diversity in Aegean Natrixnatrixmoreotica (Bedriaga, 1882) (Reptilia, Serpentes, Natricidae)
Source: Zookeys. 2023 Jul 13;1169:87–94. doi: 10.3897/zookeys.1169.104594 (PMC10846685; doi:10.3897/zookeys.1169.104594)
Supplement: Supplementary material 1 — Supplementary information [file zookeys-1169-087_article-104594__-s001.pdf]

ZooKeys
A peer-reviewed open-access journal
Launched to accelerate biodiversity research

Supplementary Information for

Genotyping the phenotypic diversity in Aegean *Natrix natrix moreotica* (Bedriaga, 1882) (Reptilia, Serpentes, Natricidae)

Daniel Jablonski, Elias Tzoras, Alexios Panagiotopoulos, Marika Asztalos, Uwe Fritz

Figure S1. Habitats of grass snakes on Milos: (**A**) collection site of *schweizeri* morphotype, (**B**) collection site of *schweizeri* and *picturata* morphotypes. Coastal habitats on Skyros: (**C**) collection site of *persa* and *picturata* morphotypes, and (**D**) habitat of the melanistic *persa* morphotype with phrygana vegetation. Photos: D. Jablonski (A) and E. Tzoras (B–D).

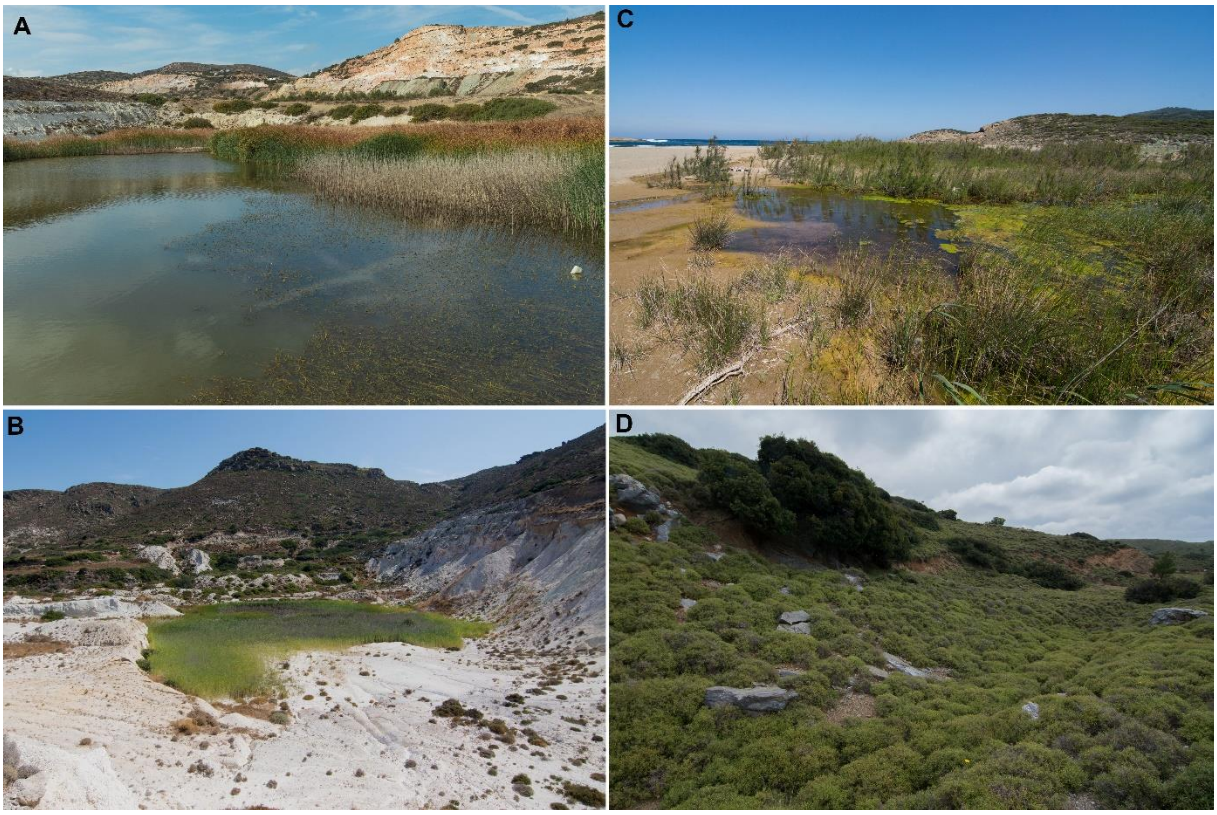

Table S1. Literature and citizen science records of the *picturata* and *schweizeri* morphotypes of *Natrix natrix* in the Aegean region and Cyprus.

Morphotype	Site	Country	N	E	Source	Link
picturata	Gouri	Cyprus	34.9576	33.1582	Zotos et al. (2021)	—
picturata	Filani Lake	Cyprus	34.9668	33.2047	Zotos et al. (2021)	—
picturata	Xyliatos Dam	Cyprus	35.0376	33.0381	Baier et al. (2009)	—
picturata	Mykonos, Cyclades	Greece	37.4462	25.3774	Cattaneo (2010)	—
picturata	Vouprasio, Peloponnese	Greece	38.0810	21.3818	Tzoras pers. observation	—
picturata	Kalamaris Waterfall, Peloponnese	Greece	36.9579	21.7305	iNaturalist; Fritz and Ihlow (2022)	https://www.inaturalist.org/observations/89532857
picturata	Voulkaria Lake	Greece	38.8680	20.8190	iNaturalist; Fritz and Ihlow (2022)	https://www.inaturalist.org/observations/36132852
picturata	Vicinity of Tsivlos Lake, Peloponnese	Greece	38.0693	22.2395	iNaturalist	https://www.inaturalist.org/observations/112552795
schweizeri	Xyliatos Dam	Cyprus	35.0376	33.0381	Baier et al. (2009)	—
schweizeri	Paralimni	Cyprus	35.0334	33.9834	Baier et al. (2009)	—
schweizeri	Karpathos, Dodecanese	Greece	35.7455	27.1767	Bogaerts et al. (2018)	—
schweizeri	Mykonos, Cyclades	Greece	37.4502	25.3885	Cattaneo (2010)	—
schweizeri	Skyros, Sporades	Greece	38.8125	24.5956	Cattaneo (2010)	—
schweizeri	Andros, Cyclades	Greece	37.8502	24.8804	Oefinger and Oefinger (2022)	https://www.eurolizards.com/trip-reports/andros-9-17-4-2022/
schweizeri	Lesvos	Greece	39.0003	26.5357	Zevgolis and Christopoulos (2023)	—
schweizeri	Argolis	Greece	37.6516	22.8582	iNaturalist; Fritz and Ihlow (2022)	https://www.inaturalist.org/observations/82552190
schweizeri	Vicinity of Vasiliko, Peloponnese	Greece	38.1184	21.7042	iNaturalist	https://www.inaturalist.org/observations/140127705
schweizeri	Vicinity of artificial Lake Aoou	Greece	39.8340	21.1611	iNaturalist; Fritz and Ihlow (2022)	https://www.inaturalist.org/observations/30259961
schweizeri	Delfoi (Delphi)	Greece	38.4821	22.5034	iNaturalist; Fritz and Ihlow (2022)	https://www.inaturalist.org/observations/26375344
schweizeri	Ikaria	Greece	37.5946	26.1218	iNaturalist; Fritz and Ihlow (2022)	https://www.inaturalist.org/observations/32329309
schweizeri	Kithira, Pireus	Greece	36.3416	22.9481	iNaturalist; Fritz and Ihlow (2022)	https://www.inaturalist.org/observations/71442789
schweizeri	Lefkada, Apollonion	Greece	38.7058	20.6037	iNaturalist; Fritz and Ihlow (2022)	https://www.inaturalist.org/observations/53348871

References

Baier F, Sparrow DJ, Wiedl HJ (2009) The Amphibians and Reptiles of Cyprus. Edition Chimaira, Frankfurt am Main, 364 pp.

Bogaerts S, Pasmans F, Protopapas D, Pafilis P, Lymberakis P (2018) Rediscovery of the grass snake (*Natrix natrix*) on the island of Karpathos, Greece. Herpetology Notes 11: 303–305.

Cattaneo A (2010) Note eco-morfologiche su alcune specie ofidiche egee, con particolare riferimento alle popolazioni delle Cicladi centro-orientali (Reptilia). Naturalista Siciliano 34: 319–350.

Fritz U, Ihlow F (2022) Citizen Science, taxonomy and grass snakes: iNaturalist helps to clarify variation of coloration and pattern in *Natrix natrix* subspecies. Vertebrate Zoology 72: 533–549.
https://doi.org/10.3897/vz.72.e87426

Zevgolis YG, Christopoulos A (2023) Entrapped in olive-harvesting nets: A case of a grass snake *Natrix natrix* from an olive-growing Greek Aegean Island. Diversity 15: 452.
https://doi.org/10.3390/d15030452

Zotos S, Stamatiou M, Naziri A, Meletiou S, Demosthenous S, Perikleous K, Erotokritou E, Xenophontos M, Zavrou D, Michael K, Sergides L (2021) New evidence on the distribution of the highly endangered *Natrix natrix cypriaca* and implications for its conservation. Animals 11: 1–13.
https://doi.org/10.3390/ani11041077
